# Serum Matrix Metalloproteinases and Left Atrial Remodeling—The Hoorn Study

**DOI:** 10.3390/ijms21144944

**Published:** 2020-07-13

**Authors:** Pauline B. C. Linssen, Hans-Peter Brunner-La Rocca, Casper G. Schalkwijk, Joline W. J. Beulens, Petra J. M. Elders, Amber A. van der Heijden, Roderick C. Slieker, Coen D. A. Stehouwer, Ronald M. A. Henry

**Affiliations:** 1 Cardiovascular Research Institute Maastricht, Maastricht University Medical Centre, 6200 MD Maastricht, The Netherlands; paulinelinssen@gmail.com (P.B.C.L.); hp.brunnerlarocca@mumc.nl (H.-P.B.-L.R.); c.schalkwijk@maastrichtuniversity.nl (C.G.S.); cda.stehouwer@mumc.nl (C.D.A.S.); 2 Department of Internal Medicine, Maastricht University Medical Centre, 6202 AZ Maastricht, The Netherlands; 3 Heart and Vascular Centre, Maastricht University Medical Centre, 6202 AZ Maastricht, The Netherlands; 4 Department of Cardiology, Maastricht University Medical Centre, 6202 AZ Maastricht, The Netherlands; 5 Department of Epidemiology and Biostatistics, Amsterdam Public Health Research Institute, Amsterdam UMC, 1007 MB Amsterdam, The Netherlands; j.beulens@amsterdamumc.nl (J.W.J.B.); r.slieker@amsterdamumc.nl (R.C.S.); 6 Julius Center for Health Sciences and Primary Care, University Medical Center Utrecht, 3508 GA Utrecht, The Netherlands; 7 Department of General Practice and Elderly Care Medicine, Amsterdam Public Health Research Institute, Amsterdam UMC, 1007 MB Amsterdam, The Netherlands; p.elders@amsterdamumc.nl (P.J.M.E.); a.vanderheijden@amsterdamumc.nl (A.A.v.d.H.); 8 Department of Cell and Chemical Biology, Leiden University Medical Center, 2300 RC Leiden, The Netherlands

**Keywords:** matrix metalloproteinase (MMPs), tissue inhibitor of matrix metalloproteinases (TIMP-1), LA-volume index, diastolic dysfunction, echocardiography

## Abstract

Extracellular matrix protein turnover may play an important role in left atrial (LA) remodelling. The aim is to investigate the associations between matrix metalloproteinase (MMPs), tissue inhibitor of metalloproteinase (TIMP-1) and LA volume index (LAVI) and if these associations are independent of TIMP-1 levels. Participants from The Hoorn Study, a population-based cohort study (*n* = 674), underwent echocardiography. Serum MMPs (i.e., MMP-1, MMP-2, MMP-3, MMP-9, and MMP-10) and TIMP-1 levels were measured with ELISA. Multiple linear regression analyses were used. MMP-1 levels were not associated with LAVI. Higher MMP-2 levels were associated with larger LAVI (regression coefficient per SD increase in MMP (95% CI); 0.03 (0.01; 0.05). Higher MMP-3 and MMP-9 levels were associated with smaller LAVI; −0.04 (−0.07; −0.01) and −0.04 (−0.06; −0.02) respectively. Only in women were higher MMP-10 levels associated with larger LAVI; 0.04 (0.00; 0.07, *p*-interaction 0.04). Additionally, only in women were higher TIMP-1 levels associated with smaller LAVI; −0.05 (−0.09; −0.01, *p*-interaction 0.03). The associations between MMPs and LAVI were independent of TIMP-1 levels. In conclusion, serum MMPs are associated with LAVI, independent of CVD risk factors and TIMP-1 levels. In addition, these associations differ according to sex and within MMP subgroups. This shows that the role of MMPs in LA remodelling is complex.

## 1. Introduction

Left atrial (LA) enlargement, as a structural consequence of diastolic dysfunction, has prognostic importance with regard to incident heart failure [1] and cardiac mortality [2]. The process of LA remodelling, which refers to the alterations in LA myocardial structure due to metabolic and hemodynamic pathobiological stimuli, is incompletely understood, although the process of extracellular matrix protein turnover plays an important role [3].

LA remodelling is characterized by the degradation of myocardial extracellular matrix (ECM) proteins orchestrated by, amongst others, a family of endopeptidases called matrix metalloproteinases (MMPs) and their endogenous inhibitors, called tissue inhibitor of metalloproteinases (TIMPs) [4,5,6]. Based on ECM substrate specificity, MMPs are subdivided into collagenases (e.g., MMP-1), gelatinases (e.g., MMP-2, MMP-9) and stromelysins (e.g., MMP-3, MMP-10) [4,5,6]. However, the function of myocardial MMPs is more complex than straightforward ECM degradation alone, as non-ECM proteins are recognized as MMP substrates as well [5]. In addition, MMP expression is also regulated by sex hormones [7,8,9]. Hence, the role of MMPs in LA remodelling may not be deducted from the process of ECM degradation only.

Previous studies [10,11,12,13,14,15,16,17,18,19,20,21] on the association between circulating MMPs and LA structure have yielded heterogeneous results. For example, some studies [10,15,17,20,21], but not all [12,14,16], showed that higher circulating MMP-2 levels were associated with greater LA dimensions. Similar, higher circulating TIMP-1 levels were associated with greater LA dimensions [12,17], whereas in other studies, these were associated with smaller LA dimensions [11,16], or were not associated with LA dimension at all [19]. Yet, most studies were done in selected populations (e.g., patients with heart failure [10,15,17,20,21], atrial fibrillation [13,18] or primary aldosteronism [12,14]), reported on a single MMP (i.e., MMP-2 [15,20,21], MMP-9 [13,18] and TIMP-1 [19]) and did not adjust for potential confounders [10,11,13,16,17,18,21]. Moreover, no previous studies reported on the associations between the stromelysins (e.g., MMP-3, MMP-10) and LA structure.

In view of the above we investigated the cross-sectional associations between serum MMPs (i.e., MMP-1, MMP-2, MMP-3, MMP-9 and MMP-10), TIMP-1 and LA volume index in population-based cohort. In addition, we investigated whether these associations, if any, were independent of TIMP-1 levels.

## 2. Results 

### 2.1. Study Population

**Figure 1:**
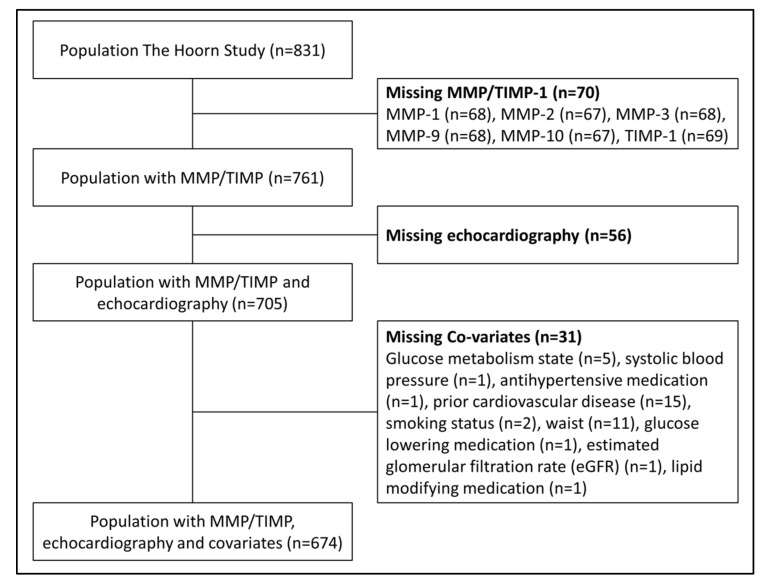
Selection of study population. Abbreviations: MMP; matrix metalloproteinase, TIMP; tissue inhibitor metalloproteinase.

For the present analyses individuals with missing data on serum MMPs or TIMP-1 (*n* = 70), echocardiographic measurements (*n* = 56) and covariates (*n* = 31) were excluded (Figure 1). The final study population therefore consisted of 674 individuals. Individuals with missing data suffered more frequently from type 2 diabetes mellitus (T2DM), more often used antihypertensive medication, had a higher waist circumference, worse kidney function (i.e., more often albuminuria and lower estimated glomerular filtration rate (eGFR)) and a less favorable lipid profile (i.e., higher levels of triglycerides and lower levels of high-density lipoprotein cholesterol). In addition, individuals with missing data had higher levels of MMP-2, MMP-9 and TIMP-1 (Supplementary Table S1).

### 2.2. Characteristics of the Study Population According to Tertiles of LA Volume Index

With increasing tertiles of LA volume index, individuals were in general older, suffered more frequently from hypertension and used more antihypertensive medication. These individuals had lower levels of triglycerides, and total and low-density-lipoprotein cholesterol. In addition, they more often had T2DM, prior cardiovascular disease (CVD), atrial fibrillation and albuminuria. Increasing tertiles of LA volume index were associated with lower left ventricular (LV) ejection fraction, greater LV end diastolic diameter, greater interventricular septal and posterior wall thicknesses and a greater LV mass index (Table 1). 

### 2.3. Association between Serum MMPs and TIMP-1 and LA Volume Index

Associations of serum MMPs and TIMP-1 with Ln LA volume index are presented in Table 2. Higher serum MMP-2 levels were (independently of age sex and glucose metabolism status (GMS)) associated with larger naturally log-transformed (Ln) LA volume index (regression coefficient per SD increase in MMP (95%CI): 0.04 (0.01; 0.06). Additional adjustments for CVD risk factors did not materially alter this association: 0.03 (0.01; 0.05). Higher serum MMP-3 and MMP-9 levels were (independent of age, sex and GMS) associated with smaller Ln LA volume index; −0.03 (−0.06; 0.00) and −0.04 (−0.06; −0.02) respectively. Additional adjustments for CVD risk factors did not materially alter these associations for serum MMP-3 and MMP-9 levels; −0.04 (−0.07; −0.01) and -0.04 (−0.06; −0.02) respectively (Table 2).

### 2.4. Stratification According to Sex

The associations of serum MMP-1, MMP-10 and TIMP-1 levels with Ln LA volume index showed significant interaction with sex. Therefore, these analyses were stratified according to sex (Table 2). Only in women were higher serum MMP-10 levels (independent of age and GMS) associated with larger Ln LA volume index; 0.03 (0.00; 0.07), *p*-interaction 0.07. Additional adjustments for CVD risk factors did not materially alter this association 0.04 (0.00; 0.07), *p*-interaction 0.04. With regard to serum TIMP-1 levels, only in women were higher serum TIMP-1 levels (independent of age, sex and GMS) associated with smaller Ln LA volume index; −0.04 (−0.08; −0.00), *p*-interaction 0.10. Additional adjustments for CVD risk factors did not materially alter this association; −0.05 (−0.09; −0.01), *p*-interaction 0.03. With regard to serum MMP-1 levels, although significant interaction between serum MMP-1 levels and sex was observed (*p*-interaction 0.09 and 0.06 for model 1 and 2 respectively), neither in women nor in men were serum MMP-1 levels associated with Ln LA volume index (Table 2).

### 2.5. TIMP-1

The associations of the individual serum MMP levels with Ln LA volume index were not materially changed after additional adjustment for serum TIMP-1 levels (Table 2, models 3).

### 2.6. Additional Analyses

No clear pattern of interaction was observed between serum MMP or TIMP-1 levels and GMS (Supplementary Table S2). Exclusion of individuals with atrial fibrillation did not change the results, except for the association of serum MMP-2 levels with Ln LA volume index, which was attenuated (Supplementary Table S3). Exclusion of individuals with wall motion abnormalities did not change the results, except for the associations of serum MMP-2 levels with Ln LA volume index, and serum MMP-10 levels with Ln LA volume index in women, which both were attenuated (Supplementary Table S4). Replacement of waist circumference by body mass index or additional adjustment for renin-angiotensin system inhibitors did not change the results (Supplementary Tables S5 and S6). Exclusion of individuals with outlier MMP/TIMP-1 values (defined as standard deviation > 3 or <−3) did not change the results, although the interaction between sex and serum TIMP-1 levels in the association with Ln LA volume index became non-significant (Supplementary Table S7).

## 3. Discussion

The present study investigated the association between an extensive set of serum MMP and LA volume index in a well-characterized population-based cohort study and has three main findings. First, higher serum MMP-2 levels were associated with greater LA volume index, whereas higher serum MMP-3 and MMP-9 levels were associated with smaller LA volume index, independent of CVD risk factors. Second, associations of MMP-10 and TIMP-1 with LA volume index differ significantly according to sex to such an extent that higher serum MMP-10 levels were associated with greater LA volume index and higher serum TIMP-1 levels were associated with smaller LA volume index only in women. Third, these associations were independent of TIMP-1 levels. Taken together, the results of this study shows that serum MMPs are associated with LA volume index, independent TIMP-1 levels and cardiovascular risk factors, although these associations differ according to sex and within MMP subgroups.

The present study extends previous research because of its population-based design; the measurement of an extensive set of serum MMPs (including MMP-3 and MMP-10) and TIMP-1, which enabled the comparison of associations within MMP subgroups and the thorough clinical characterization of its participants, which enabled an extensive adjustment for potential confounders. With regard to serum MMP-2, in most previous research [10,15,17,20,21], a similar positive association of MMP-2 levels with LA volume was observed, although smaller studies [12,14,16] and/or performed in patients with primary aldosteronism [12,14] did not observe this association. With regard to serum MMP-9, in contrast to previous research, which did observe a positive association of MMP-9 levels with LA volume [10,11,18], or no association [13,14,17], we observed a negative association. These studies were, however, performed in selected populations (i.e., patients with heart failure [10,17], atrial fibrillation [13,18] or primary aldosteronism [14]). The positive association of MMP-9 with LA volume in the longitudinal study of Collier et al [11], with a comparable study population (i.e., patients with known risk factors for heart failure) may be due to a selection bias, as this study excluded patients with a decreasing LA volume. With regard to serum TIMP-1, previous study results have been heterogeneous [11,12,16,17,19]. Only Sundström et al investigated effect modification by sex, which was not present, in contrast to our study, although in this study, LA diameter, instead of volume, was measured, which is not necessarily representative of LA enlargement in LA remodeling [22].

The function of matrix metalloproteinases is primarily ECM degradation. In this context, our results showed contrasting results within subgroup of MMPs (i.e., based on ECM substrate specificity). These results are supported by the in vivo work of Solomonov et al. [23], who showed that structurally homologous collagenases (i.e., MMP-1 and MMP-13), in contrast to in vitro studies, cause different ECM degradation patterns with consequential different alterations in tissue morphology, viscoelastic and biochemical properties. Hence, MMP activity is influenced by microenvironment and not substrate specificity alone [23,24]. Against this background, the interplay between ECM remodeling and immunological pathways is also relevant: secreted cytokines and growth factors are bound in the ECM, and consequently ECM degradation has immunomodulatory activity; cytokines and growth factors can stimulate the expression of MMPs itself (e.g., TGF-β, IL-1β, and TNF-α). Furthermore, ECM breakdown products, so-called matrikines, can have chemoattractant properties [25,26]. This interplay is illustrated by the study of Egerstedt et al., in which proteomic analyses across different stages of human heart failure show both the importance of matrix remodeling as well as the immune system [27]. Taken together, it seems that the relation of MMPs with LA remodeling cannot be deduced from only ECM degradation and is more complex [28]. 

Our results also showed sex-related differences in the association between serum MMPs and LA remodeling. Previous studies already showed different patterns of cardiac remodeling between sex, which could be related to MMP [8,29,30,31]. For instance, Montalvo et al. [7] showed, in a pressure-overloaded mice model, that myocardial fibrosis was increased in male mice, due to myocardial TGF-β induction by gonadal androgens, which could be prevented by orchidectomy or inhibition of TGF-β. TGF-β itself regulates MMP and TIMP activity [26]. The role of androgens was further emphasized by Coronado et al. [8], who showed that myocardial fibrosis was induced by androgens (via IL-1β and serpin A 3n) by modulating MMPs and TIMP-1 expression. Moreover, Voloshenyuk et al [32] showed in rats with volume overloaded hearts and ovariectomy, that estrogen depletion exacerbated cardiac remodeling and was associated with TIMP/MMP imbalance, which was prevented by estrogen supplementation. Hence, both sex hormones may contribute to the sex related differences in the association between serum MMPs and LA remodeling. 

Certain limitations of the present study need to be taken into consideration. Firstly, we used serum MMPs and TIMP-1 levels, which may not necessarily reflect myocardial interstitial levels. Although previous research showed a good correlation between circulating MMP levels and myocardial tissue MMP levels [33,34], MMPs are activated locally and it remains unclear whether serum levels reflect local MMP activity [4]. In similar vein, serum TIMP-1 levels may not be reflective of local inhibition by TIMP-1. Secondly, in this study, serum samples were used to measure MMPs and TIMP-1. Due to the absolute differences in plasma versus serum MMPs levels [35,36,37,38,39], these results may not be directly extrapolated. However, previous work in different cohorts with both plasma and serum samples showed that associations were largely consistent throughout these cohort [40]. Thirdly, absolute levels of MMP-9 decrease over time. Hence, due to prolonged storage time, absolute MMP-9 levels are low. However, this decrease can be considered similar for all blood samples and therefore is not likely to have affected the main results. Fourthly, the cross-sectional design does not allow us to make any strong causal inferences. Finally, the excluded study population differed mainly with regard to worse cardiac risk profile, but we note that adjustments for CVD risk factors were of limited impact. We therefore think that this issue is unlikely to have affected the main results of these analyses. In addition, this study was performed in a middle-aged to elderly Caucasian population; the results of this study may not be directly translated to other study populations.

## 4. Materials and Methods 

### 4.1. Study Population

For the current analyses, we used data from The Hoorn Study, an observational population based cohort study of GMS in relation to CVD risk factors [41,42]. In 1989, 2484 individuals, aged 50–75 years, from the population register of the Dutch town of Hoorn participated in the baseline examination. In 1996–1998, 1513 (73%) of all surviving participants agreed to participate in a follow-up visit. In 2000–2001, all those who were diagnosed as having diabetes during previous examination (*n* = 176) and random samples of individuals with normal glucose metabolism (*n* = 705) and intermediate hyperglycaemia (*n* = 193) were invited, of whom 648 (60%) participated. To increase the number of individuals with T2DM, 183 individuals with T2DM from The Hoorn Screening Study were added. The total study population therefore consisted of 831 individuals. The study has been complied with the Declaration of Helsinki, the Medical Ethical Committee of the VU University Medical Centre, Amsterdam, The Netherlands has approved the research protocol (89-71 22 June 1989 and 99-168 10 November 1999) and informed consent has been obtained [41].

### 4.2. Measurement of Serum MMPs and TIMP-1

Levels of MMPs and TIMP-1 were measured in serum samples obtained from fasting venous blood, which were stored at −80 °C. Serum levels of MMPs and TIMP-1 were measured by multi-array electrochemiluninescence platforms of MesoScaleDiscovery (MSD, Gaithersburg, MD, USA) with the use of commercially available kits (Human MMP-3-Plex Kit for MMP-1,-3,-9; Human MMP-2-Plex Kit for MMP-2, -10; Human TIMP-1 Kit for TIMP-1: MSD, Gaithersburg, MD, USA) according to the manufacturer’s protocol. The MMPs were detected in both their pro- and active forms, whereas TIMP-1 was detected only in its active form. Intra- and inter-assay coefficients of variation were 7.0% and 8.0% for MMP-1, 4.5% and 5.9% for MMP-2, 8.4% and 12.3% for MMP-3, 5.3% and 8.9% for MMP-9, 4.4% and 9.7% for MMP-10 and 4.3% and 5.2% for TIMP-1, respectively. Data collection took place in 2000–2001. MMP and TIMP-1 levels were measured in 2012.

### 4.3. Echocardiography

Echocardiography was performed according to a standardized protocol consisting of two dimensional and M-mode assessments [43] with the use of a Hewlett Packard SONOS 5500 echocardiography system (2-4 MHz transducer, Andover, MA, USA). LA volume was calculated from the apical four chamber view with the use of the modified Simpson formula and indexed to body surface area [22,44]. M-mode measurements of interventricular septum thickness (IVS), posterior wall thickness (PWT) and LV end-diastolic diameter (LVEDD) were obtained according to the recommendations of the American Society of Echocardiography [22,44]. LV mass was calculated as 0.8 × 1.04 × (((LVEDD + IVS + PWT)^3^) – (LVEDD^3^) + 0.6) and indexed to body surface area. Systolic function was defined with the use of monoplane Simpson’s LV ejection fraction, calculated from the LV systolic and diastolic volumes in the apical four chamber view. In addition, each echocardiogram was checked for the presence of LV wall motion abnormalities.

### 4.4. Covariates

GMS was defined according to the 1999 WHO criteria [45] with an oral glucose tolerance test as previously described. Intermediate hyperglycaemia was defined as impaired fasting glucose and/or impaired glucose tolerance (i.e., fasting plasma glucose level 6.1–6.9 mmol/L, or post-load glucose level 7.8–11.0 mmol/L 2 h after 75 g oral glucose tolerance test, respectively). Systolic pressure, use of antihypertensive medication, prevalent CVD, current smoking, waist circumference, use of glucose lowering medication, eGFR [46], presence of albuminuria (defined as urinary albumin excretion ≥ 30 mg/24 h) [47], total cholesterol, high-density lipoprotein cholesterol, triglycerides, use of lipid-modifying medication and use renin-angiotensin system modifying agents (defined as angiotensin converting enzyme inhibitors, angiotensin II blockers, renin inhibitors and/or aldosterone antagonists) were determined as previously described [42,48].

### 4.5. Statistical Analyses

All analyses were performed with the statistical software package SPSS version 23.0 (IBM Corp, Armonk, NY, USA). Descriptive statistics are presented as mean (±standard deviation), or in case of a skewed distribution as median (interquartile range), or frequencies (percentages). Variables with a skewed distribution were naturally log-transformed in order to meet normality criteria. For reasons of direct comparison of serum MMP and TIMP-1, *Z*-scores (individual value − mean_population_)/(standard deviation_population_) were calculated. We used linear regression analyses to investigate the associations between Z-scores of the individual serum MMPs and TIMP-1 and LA volume index. Adjustments were made first for age, sex and GMS (model 1). Additional adjustments were made for known CVD risk factors (i.e., systolic pressure, the use of antihypertensive medication, current smoking, waist circumference, glucose-lowering medication (including insulin), eGFR, albuminuria, total cholesterol, high-density lipoprotein cholesterol, triglycerides, lipid-modifying medication and the presence of prior CVD) (model 2). To test whether the associations between MMPs and LA volume index were independent of any presumable inhibitory effects by TIMP-1, additional adjustments were made for TIMP-1 levels (model 3). Interaction terms were used to investigate whether the association between MMPs and LA volume index differed according to sex or GMS. *p*-values < 0.05 were considered statistically significant, except for the interaction analyses where *p*-values < 0.10 were used.

## 5. Conclusions

In conclusion, serum MMPs are associated with LA volume index, independent of TIMP-1 levels and CVD risk factors. These associations differ according to sex and within MMP subgroups. This shows that the role of MMPs in LA remodeling is complex. Future research, for example proteomic studies or studies with selective MMP inhibitors, should give more insight into the regulation of MMPs activity and effects of MMP in progression of cardiac disease.

## Figures and Tables

**Table 1:** Clinical characteristics of the study population according to tertiles of left atrial volume index (*n* = 674).

Demographics	Lowest Tertile (*n* = 224)		Middle Tertile (*n* = 225)		Highest Tertile (*n* = 225)		*p*_trend_
Women, %	105	(46.9)	113	(50.2)	118	(52.4)	0.24 ^†^
Age, years	67.4	±6.1	67.9	±6.5	69.8	±7.9	<0.01
**Obesity**							
BMI, kg/m^2^	27.3	±3.6	27.6	±3.9	27.1	±3.6	0.55
Waist, cm	95.3	±11.1	95.3	±11.1	95.2	±11.6	0.95
**Blood pressure**							
Systolic blood pressure	140.5	±18.9	139.8	±19.8	144.0	±21.9	0.06
Diastolic blood pressure, mmHg	83.4	±10.4	83.4	±11.1	82.7	±11.2	0.46
Hypertension	143	(63.8)	149	(66.2)	171	(76.0)	<0.01 ^†^
Antihypertensive medication	57	(25.4)	79	(35.1)	113	(50.2)	<0.01 ^†^
RAS inhibitors	19	(8.5)	22	(9.8)	39	(17.3)	<0.01 ^†^
**Cholesterol**							
Total cholesterol, mmol/L	5.8	±1.1	5.7	±1.0	5.6	±1.0	0.02
HDL cholesterol, mmol/L	1.39	±0.40	1.40	±0.39	1.44	±0.44	0.18
LDL cholesterol, mmol/L	3.7 ^a^	±1.0	3.7 ^b^	±0.9	3.5	±0.9	0.03
Triglycerides, mmol/L	1.4	(1.0–2.0)	1.3	(1.0–1.8)	1.3	(0.9–1.8)	<0.01
Lipid modifying medication	37	(16.5)	41	(18.2)	34	(15.1)	0.69 ^†^
**Glucose metabolism**							
HbA1c, %	6.03 ^b^	±0.63	6.01	±0.82	6.03	±0.70	0.99
Fasting plasma glucose, mmol/L	6.24	±1.40	6.29	±1.51	6.48	±1.44	0.09
Glucose lowering medication	8	(3.6)	13	(5.8)	16	(7.1)	0.10 ^†^
**Glucose metabolism status**							
Normal	100	(44.6)	101	(44.9)	72	(32.0)	<0.01 ^†^
Intermediate hyperglycaemia	71	(31.7)	49	(21.8)	49	(21.8)	
Type 2 diabetes mellitus	53	(23.7)	75	(33.3)	104	(46.2)	
**Other CVD risk factors**							
Prior CVD	92	(41.1)	108	(48.0)	120	(53.3)	0.01 ^†^
Atrial fibrillation	0	(0.0)	0	(0.0)	16 ^b^	(7.1)	<0.01 ^†^
Current smoking	40	(17.9)	31	(13.8)	28	(12.4)	0.10 ^†^
Kidney function							
albuminuria	17	(7.6)	14	(6.2)	37	(16.4)	<0.01 ^†^
eGFR (mL/min/1.73 m^2^)	64.2	±10.6	65.8	±9.7	64.9	±11.4	0.53
**Echocardiographic data**							
LA volume index, mL/m^2^	17.4	(15.9–18.7)	22.0	(21.0–23.4)	29.9	(26.8–34.5)	*
LV ejection fraction, % ^a^	62.8 ^c^	±7.3	62.4 ^d^	±7.2	59.7 ^d^	±9.7	<0.01
LV end diastolic diameter, mm	49.7 ^a^	±5.2	50.2 ^b^	±5.7	52.1	±6.4	<0.01
Inter ventricular septum, mm	9.4 ^b^	±2.1	9.6 ^b^	±2.2	10.3	±2.9	<0.01
Posterior wall thickness, mm	8.8 ^a^	±1.4	8.9 ^b^	±1.4	9.3	±1.7	<0.01
LV mass index, gr/m^2^	83 ^a^	±20	88 ^b^	±22	101	±31	<0.01
E/A ratio	0.81	± 0.19	0.84	±0.23	0.88	±0.35	<0.01
Wall motion abnormalities	10	(4.5)	12	(5.3)	22	(9.8)	0.05
**Matrix metalloproteinases**							
MMP-1, ng/mL	11.3	(5.5–19.3)	10.9	(6.4–21.3)	12.7	(6.8–21.9)	0.22
MMP-2, ng/mL	96	(88–108)	98	(89–109)	104	(91–114)	<0.01
MMP-3, ng/mL	12.3	(7.8–18.3)	10.5	(7.2–15.8)	10.5	(7.0–15.4)	0.09
MMP-9, ng/mL	54	(34–85)	49	(31–79)	46	(31–73)	<0.01
MMP-10, pg/mL	831	(608–1243)	838	(616–1119)	852	(638–1232)	0.88
TIMP-1, ng/mL	321	(268–370)	302	(266–346)	307	(262–347)	0.10

Data are presented as frequencies (percentages), means ± standard deviation or median (interquartile range). Abbreviations: BMI; body mass index, HDL; high density lipoprotein, LDL; low density lipoprotein, RAS; renin-angiotensin-system, HbA1c; haemoglobin A1c, CVD; cardiovascular disease, eGFR; estimated glomerular filtration rate, MMP: matrix metalloproteinase, TIMP: tissue inhibitor metalloproteinase, LA; left atrial, LV; left ventricular. Numbers of missing data: ^a^ *n* = 2, ^b^ *n* = 1, ^c^ *n* = 13, ^d^ *n* = 8 * Not applicable ^†^ *p*-value Chi-square.

**Table 2:** Associations of serum MMPs and TIMP-1 with Ln left atrial volume index: overall and stratified to sex (linear regression, *n* = 674).

	Model	Overall	Men	Women	*p*-interaction
		β (95% CI)	β (95% CI)	β (95% CI)	Sex and MMP/TIMP
MMP-1 (SD)	1	0.01 (−0.02; 0.03)	0.03 (0.00; 0.06)	−0.01 (−0.04; 0.02)	0.09
	2	0.00 (−0.02; 0.02)	0.02 (−0.01; 0.05)	−0.02 (−0.05; 0.01)	0.06
	3	0.01 (−0.02; 0.03)	0.03 (0.00; 0.06)	−0.02 (−0.05; 0.02)	0.05
MMP-2 (SD)	1	0.04 (0.01; 0.06) †	-	-	0.58
	2	0.03 (0.01; 0.05) *	-	-	0.45
	3	0.03 (0.00; 0.05) *	-	-	0.46
MMP-3 (SD)	1	−0.03 (−0.06; 0.00) *	-	-	0.97
	2	−0.04 (−0.07; −0.10) †	-	-	0.84
	3	−0.04 (−0.07; −0.01) *	-	-	0.86
MMP-9 (SD)	1	−0.04 (−0.06; −0.02) †	-	-	0.47
	2	−0.04 (−0.06; −0.02) †	-	-	0.46
	3	−0.04 (−0.06; −0.02) †	-	-	0.46
MMP-10 (SD)	1	0.01 (−0.01; 0.03)	−0.01 (−0.04; 0.02)	0.03 (0.00; 0.07) *	0.07
	2	0.01 (−0.01; 0.03)	−0.01 (−0.04; 0.02)	0.04 (0.00; 0.07) *	0.04
	3	0.01 (−0.01; 0.04)	−0.01 (−0.04; 0.03)	0.04 (0.01; 0.07) *	0.05
TIMP-1 (SD)	1	−0.01 (−0.04; 0.01)	0.00 (−0.03; 0.03)	−0.04 (−0.08; 0.00) *	0.10
	2	−0.02 (−0.04; 0.01)	0.00 (−0.03; 0.03)	−0.05 (−0.09; −0.01) †	0.03

Model 1: adjusted for age, sex (as appropriate), glucose metabolism status. Model 2: model 1 + systolic blood pressure, use of antihypertensive medication, prior cardiovascular disease, current smoking, waist, use of glucose- lowering medication (including insulin), estimated glomerular filtration rate, presence of albuminuria, total cholesterol, high density lipoprotein cholesterol, Ln triglycerides, use of lipid- modifying medication Model 3: model 2 + TIMP-1. *p* value * < 0.05, † < 0.01

## Supplementary Materials

Supplementary materials can be found at https://www.mdpi.com/1422-0067/21/14/4944/s1.
