# Supplementary material for: Serum Matrix Metalloproteinases and Left Atrial Remodeling—The Hoorn Study
Source: Int J Mol Sci. 2020 Jul 13;21(14):4944. doi: 10.3390/ijms21144944 (PMC7404388; doi:10.3390/ijms21144944)
Supplement: Supplementary file 1 [file ijms-21-04944-s001.pdf]

SUPPLEMENTAL TABLES

Serum matrix metalloproteinases and left atrial remodelling -The Hoorn Study-

Pauline B.C. Linssen MD, Hans-Peter Brunner-La Rocca MD, Casper G. Schalkwijk, Joline Beulens, Petra J.M. Elders, Amber van der Heijden, Roderick C. Slieker, Coen D.A. Stehouwer, Ronald M.A. Henry

TABLES

Supplemental table S1: Clinical characteristics of the included versus excluded study population

	Included (n=674)	Excluded (n=157)	P
Demographics			
Women,%	336 (49.9)	83 (52.9)	0.50
Age, years	68.4 ±6.9	69.5 ±8.3	0.07
Obesity			
BMI, kg/m2	27.3 ±3.7	29.8 ±5.7[e]	<0.01
Waist, cm	95.2 ±11.3	102.4 ±14.6[f]	<0.01
Blood pressure			
Systolic blood pressure	141.4 ±20.3	144.9 ±19.9[g]	0.07
Diastolic blood pressure, mmHg	83.1 ±10.9	83.7 ±11.1[g]	0.56
Hypertension	463 (68.7)	99 (76.7)[h]	0.07
Antihypertensive medication	249 (36.9)	64 (48.9)[i]	0.01
RAS inhibitors	80 (11.9)	35 (22.9)[j]	<0.01
Cholesterol			
Total cholesterol, mmol/l	5.7 ±1.1	5.6 ±0.9[h]	0.285
High density lipoprotein cholesterol, mmol/l	1.4 ±0.4	1.3 ±0.3[h]	<0.01
Low density lipoprotein cholesterol, mmol/l	3.6 ±0.9[a]	3.5 ±0.8[c]	0.24
Triglycerides, mmol/l	1.3 (1.0-1.8)	1.6 (1.2-2.3)[h]	<0.01
Lipid modifying medication	112 (16.6)	20 (15.3)	0.70
Glucose metabolism			
HbA1c, %	6.0 ±0.7[b]	6.5 ±0.9[g]	<0.01
Fasting plasma glucose, mmol/l	6.3 ±1.5	7.3 ±1.6[g]	<0.01
Glucose lowering medication	37 (5.5)	22 (16.8)[i]	<0.01
Glucose metabolism status			
Normal	273 (40.5)	15 (12.4)[k]	<0.01
Intermediate hyperglycaemia	169 (25.1)	12 (9.9)	
Type 2 diabetes mellitus	232 (34.4)	94 (77.7)	
Prior cardiovascular disease	320 (47.5)	53 (51.5)[l]	0.45
Atrial fibrillation	16 (2.4)[b]	1 (0.8)[g]	0.24
Current smoking	99 (14.7)	21 (16.2)[g]	0.67
Kidney function			
albuminuria	68 (10.1)	22 (17.3)[m]	0.02
eGFR(ml/min/1.73m^2)	65.0 ±10.6	62.4 ±12.5[c]	0.01
Echocardiographic data			
LA volume index, ml/m^2	22.0 (18.7-26.9)	23.3 (19.2-27.7)[n]	0.48
LV ejection fraction, %[a]	61.6 ±8.3[c]	59.4 ±8.5[n]	0.02
LV end diastolic diameter, mm	50.7 ±5.9[a]	50.7 ±5.7[k]	0.98
Inter ventricular septum, mm	9.8 ±2.4[d]	10.2 ±2.3[k]	0.09
Posterior wall thickness, mm	9.0 ±1.5[a]	9.4 ±1.7[k]	0.02
LV mass index, gr/m^2	90.6 ±26.1[a]	94.8 ±31.8[k]	0.12
Wall motion abnormalities	44 (6.5)	9 (8.0)[o]	0.58
Matrix metalloproteinases			
MMP-1, ng/ml	11.5 (6.3-21.3)	15.2 (8.0-25.1)[p]	0.08
MMP-2, ng/ml	99 (89-111)	106 (97-117)[q]	<0.01
MMP-3, ng/ml	10.9 (7.3-16.5)	11.7 (6.9-18.9)[p]	0.344
MMP-9, ng/ml	49 (32-78)	64 (44-92)[p]	<0.01
MMP-10, pg/ml	844 620-1201)	746 (513-1188)[q]	0.12
TIMP-1, ng/ml	309 (267-352)	326 (279-370)[r]	0.02

Data are presented as frequencies (percentages), means ± standard deviation or median (interquartile range). Abbreviations: BMI; body mass index, RAS; renin-angiontensin-system, HbA1c; haemoglobin A1c, eGFR; estimated glomerular filtration rate, MMP: matrix metalloproteinase, TIMP: tissue inhibitor metalloproteinase, LA; left atrial, LV; left ventricular.
Numbers of missing data: a n=3, b n=1, c n=29, d n=2, e n=25, f n=41, g n=27, h n=28, i n=26, j n=4, k n=36, l n=54, m n=30, n n=62, o n=44, p n=68, q n=67, r n=69
*Not applicable † P-value Chi-square

Supplemental table S2: Associations of serum MMP's and TIMP-1 levels with Ln LA volume index, stratified to sex and glucose metabolism status (linear regression, n=674)

	Model	Overall β (95% CI)	$P_{int\ gms}$	$P_{int\ sex}$	Men β (95% CI)	$P_{int\ gms}$	Women β (95% CI)	$P_{int\ gms}$	NGM β (95% CI)	$P_{int\ sex}$	Intermediate hyperglycemia β (95% CI)	$P_{int\ sex}$	Diabetes β (95% CI)	$P_{int\ sex}$
MMP-1	1	0.01 (-0.02;0.03)	0.003	0.09	0.03 (0.00;0.06)	0.02	-0.01 (-0.04;0.02)	0.04	-0.04 (-0.07;0.00)	0.71	0.009 (-0.04;0.06)	0.28	0.05 (0.01; 0.08)*	0.13
(SD)	2	0.00 (-0.02;0.02)	0.004	0.06	0.02 (-0.01;0.05)	0.01	-0.02 (-0.05;0.01)	0.07	-0.04 (-0.07;0.00)*	0.57	-0.017 (-0.07;0.03)	0.56	0.03 (0.00;0.07)	0.08
	3	0.01 (-0.02;0.03)	0.003	0.05	0.03 (0.00;0.06)	0.01	-0.02 (-0.05;0.02)	0.05	-0.03 (-0.07;0.01)	0.55	-0.016 (-0.07;0.03)	0.56	0.04 (0.01;0.08)*	0.01
MMP-2	1	0.04 (0.01;0.06)†	0.33	0.58	-	-	-	-	-	-	-	-	-	-
(SD)	2	0.03 (0.01;0.05)*	0.47	0.45	-	-	-	-	-	-	-	-	-	-
	3	0.03 (0.00;0.05)*	0.48	0.46	-	-	-	-	-	-	-	-	-	-
MMP-3	1	-0.03 (-0.06;0.00)*	0.11	0.97	-	-	-	-	-	-	-	-	-	-
(SD)	2	-0.04 (-0.07;-0.10)†	0.21	0.84	-	-	-	-	-	-	-	-	-	-
	3	-0.04 (-0.07;-0.01)*	0.18	0.86	-	-	-	-	-	-	-	-	-	-
MMP-9	1	-0.04 (-0.06;-0.02)†	0.14	0.47	-	-	-	-	-	-	-	-	-	-
(SD)	2	-0.04 (-0.06;-0.02)†	0.35	0.46	-	-	-	-	-	-	-	-	-	-
	3	-0.04 (-0.06;-0.02)†	0.34	0.46	-	-	-	-	-	-	-	-	-	-
MMP-10	1	0.01 (-0.01;0.03)	0.54	0.07	-0.01 (-0.04;0.02)	0.69	0.03 (0.00;0.07)*	0.52	-	-	-	-	-	-
(SD)	2	0.01 (-0.01;0.03)	0.59	0.04	-0.01 (-0.04;0.02)	0.72	0.04 (0.00;0.07)*	0.45	-	-	-	-	-	-
	3	0.01 (-0.01;0.04)	0.59	0.05	-0.01 (-0.04;0.03)	0.72	0.04 (0.01;0.07)*	0.52	-	-	-	-	-	-
TIMP-1	1	-0.01 (-0.04;0.01)	0.14	0.10	0.00 (-0.03;0.03)	0.01	-0.04 (-0.08;0.00)*	0.34	-0.05 (-0.09;0.00)	0.22	0.001 (-0.05;0.06)	0.04	-0.01 (-0.04;0.02)	0.27
(SD)	2	-0.02 (-0.04;0.01)	0.17	0.03	0.00 (-0.03;0.03)	0.06	-0.05 (-0.09;-0.01)†	0.42	-0.04 (-0.09;0.01)	0.74	-0.008 (-0.06;0.05)	0.14	-0.01 (-0.04;0.02)	0.08

Model 1: adjusted for age, sex (as appropriate), glucose metabolism status(as appropriate)
Model 2: model 1 + systolic blood pressure, use of antihypertensive medication, prior cardiovascular disease, current smoking, waist, use of glucose- lowering medication (including insulin), estimated glomerular filtration rate, presence of albuminuria, total cholesterol, high density lipoprotein cholesterol, Ln triglycerides, use of lipid- modifying medication
Model 3: model 2 + TIMP-1
*P value *< 0.05, †<0.01*

Supplemental table S3: Associations of serum MMP's and TIMP-1 levels with Ln LA volume index, in the population without atrial fibrillation (linear regression, n=657)

	Model	Overall<br>β (95% CI)	Men<br>β (95% CI)	Women<br>β (95% CI)	$P_{interaction}$<br>sex and MMP/TIMP
MMP-1	1	0.01 (-0.01;0.03)	0.03 (0.00;0.06)*	-0.01 (-0.04;0.02)	0.05
(SD)	2	0.00 (-0.02;0.03)	0.03 (0.00;0.06)	-0.02 (-0.04;0.01)	0.04
	3	0.01 (-0.01;0.03)	0.04 (0.01;0.07)*	-0.01 (-0.04;0.02)	0.03
MMP-2	1	0.02 (0.00;0.05)*	-	-	0.17
(SD)	2	0.02 (0.00;0.04)	-	-	0.18
	3	0.02 (-0.01;0.04)	-	-	0.18
MMP-3	1	-0.02 (-0.05;0.01)	-	-	0.66
(SD)	2	-0.03 (-0.06;0.00)*	-	-	0.80
	3	-0.03 (-0.05;0.00)*	-	-	0.79
MMP-9	1	-0.03 (-0.05;-0.01)†	-	-	0.16
(SD)	2	-0.03 (-0.05;-0.01)†	-	-	0.14
	3	-0.03 (-0.05;-0.01)*	-	-	0.14
MMP-10	1	0.01 (-0.01;0.03)	-0.01 (-0.04;0.02)	0.04 (0.01;0.07)*	0.02
(SD)	2	0.01 (-0.01;0.03)	-0.01 (-0.04;0.02)	0.04 (0.01;0.07)*	0.01
	3	0.02 (-0.01;0.04)	-0.01 (-0.04;0.02)	0.04 (0.01;0.07)†	0.02
TIMP-1 (SD)	1	-0.02 (-0.04;0.01)	0.00 (-0.03;0.03)	-0.04 (-0.07;-0.01)*	0.07
	2	-0.02 (-0.04;0.01)	0.00 (-0.02;0.03)	-0.05 (-0.08;-0.02)†	0.01

Model 1: adjusted for age, sex (as appropriate), glucose metabolism status
Model 2: model 1 + systolic blood pressure, use of antihypertensive medication, prior cardiovascular disease, current smoking, waist, use of glucose- lowering medication (including insulin), estimated glomerular filtration rate, presence of albuminuria, total cholesterol, high density lipoprotein cholesterol, Ln triglycerides, use of lipid- modifying medication
Model 3: model 2 + TIMP-1
*P value *< 0.05, †<0.01*

Supplemental table S4: Associations of serum MMP's and TIMP-1 levels with Ln LA volume index, in the population without wall motion abnormalities (linear regression, n=629)

	Model	Overall<br>β (95% CI)	Men<br>β (95% CI)	Women<br>β (95% CI)	$P_{interaction}$<br>sex and MMP/TIMP
MMP-1	1	0.01 (-0.01;0.04)	0.04 (0.01;0.07)*	-0.01 (-0.04;0.02)	0.03
(SD)	2	0.01 (-0.02;0.03)	0.03 (0.00;0.07)*	-0.02 (-0.05;0.01)	0.02
	3	0.01 (-0.01;0.04)	0.04 (0.01;0.08)*	-0.01 (-0.04;0.02)	0.01
MMP-2	1	0.03 (0.01;0.05)*	-	-	0.38
(SD)	2	0.02 (0.00;0.05)	-	-	0.37
	3	0.02 (0.00;0.04)	-	-	0.37
MMP-3	1	-0.03 (-0.06;0.00)*	-	-	0.70
(SD)	2	-0.04 (-0.07;-0.01)†	-	-	0.65
	3	-0.04 (-0.07;-0.01)*	-	-	0.66
MMP-9	1	-0.03 (-0.06;-0.01)†	-	-	0.13
(SD)	2	-0.04 (-0.06;-0.01)†	-	-	0.14
	3	-0.03 (-0.06;-0.01)†	-	-	0.14
MMP-10	1	0.01 (-0.02;0.03)	-0.01 (-0.04;0.02)	0.03 (-0.01;0.06)	0.12
(SD)	2	0.01 (-0.02;0.03)	-0.01 (-0.04;0.02)	0.03 (0.00;0.06)	0.09
	3	0.01 (-0.01;0.04)	-0.01 (-0.04;0.02)	0.03 (0.00;0.06)	0.09
TIMP-1 (SD)	1	-0.02 (-0.04;0.01)	0.00 (-0.03;0.03)	-0.04 (-0.08;0.00)*	0.12
	2	-0.02 (-0.04;0.00)	0.00 (-0.03;0.03)	-0.05 (-0.08;-0.01)†	0.04

Model 1: adjusted for age, sex (as appropriate), glucose metabolism status
Model 2: model 1 + systolic blood pressure, use of antihypertensive medication, prior cardiovascular disease, current smoking, waist, use of glucose- lowering medication (including insulin), estimated glomerular filtration rate, presence of albuminuria, total cholesterol, high density lipoprotein cholesterol, Ln triglycerides, use of lipid- modifying medication
Model 3: model 2 + TIMP-1
*P value *< 0.05, †<0.01*

Supplemental table S5: Associations of serum MMP's and TIMP-1 levels with Ln LA volume index, adjustment for body mass index instead of waist (linear regression, n=674)

	Model	Overall β (95% CI)	Men β (95% CI)	Women β (95% CI)	$P_{interaction}$ sex and MMP/TIMP
MMP-1	1	0.01 (-0.02;0.03)	0.03 (-0.01;0.06)	-0.01 (-0.04;0.02)	0.09
(SD)	2	0.00 (-0.02;0.02)	0.02 (-0.01;0.05)	-0.02 (-0.05;0.01)	0.07
	3	0.01 (-0.02;0.03)	0.03 (-0.01;0.06)	-0.02 (-0.05;0.02)	0.05
MMP-2	1	0.04 (0.01;0.06)†	-	-	0.58
(SD)	2	0.03 (0.01;0.05)*	-	-	0.43
	3	0.03 (0.00;0.05)*	-	-	0.43
MMP-3	1	-0.03 (-0.06;0.00)*	-	-	0.97
(SD)	2	-0.04 (-0.07;-0.01)†	-	-	0.79
	3	-0.04 (-0.07;-0.01)†	-	-	0.81
MMP-9	1	-0.04 (-0.06;-0.02)†	-	-	0.47
(SD)	2	-0.04 (-0.06;-0.02)†	-	-	0.47
	3	-0.04 (-0.06;-0.02)†	-	-	0.47
MMP-10	1	0.01 (-0.01;0.03)	-0.01 (-0.04;0.02)	0.03 (0.00;0.07)*	0.07
(SD)	2	0.01 (-0.01;0.03)	-0.01 (-0.04;0.02)	0.04 (0.00;0.07)*	0.04
	3	0.01 (-0.01;0.04)	-0.01 (-0.04;0.02)	0.04 (0.01;0.07)*	0.05
TIMP-1 (SD)	1	-0.01 (-0.04;0.01)	0.00 (-0.03;0.03)	-0.04 (-0.08;0.00)*	0.10
	2	-0.02 (-0.04;0.01)	0.00 (-0.03;0.03)	-0.05 (-0.09;-0.01)†	0.03

Model 1: adjusted for age, sex (as appropriate), glucose metabolism status
Model 2: model 1 + systolic blood pressure, use of antihypertensive medication, prior cardiovascular disease, current smoking, body mass index, use of glucose- lowering medication (including insulin), estimated glomerular filtration rate, presence of albuminuria, total cholesterol, high density lipoprotein cholesterol, Ln triglycerides, use of lipid- modifying medication
Model 3: model 2 + TIMP-1
*P value *< 0.05, †<0.01*

Supplemental table S6: Associations of serum MMP's and TIMP-1 levels with Ln LA volume index, additional adjustment for use of renin-angiontensin-system inhibitors (linear regression, n=674)

	Model	Overall β (95% CI)	Men β (95% CI)	Women β (95% CI)	$P_{interaction}$ sex and MMP/TIMP
MMP-1	1	0.01 (-0.02;0.03)	0.03 (0.00;0.06)	-0.02 (-0.05;0.02)	0.05
(SD)	2	0.00 (-0.02;0.03)	0.03 (-0.01;0.06)	-0.02 (-0.05;0.02)	0.06
MMP-2	1	0.03 (0.00;0.05)*	-	-	0.46
(SD)	2	0.03 (0.01;0.05)*	-	-	0.57
MMP-3	1	-0.04 (-0.07;-0.01)*	-	-	0.86
(SD)	2	-0.04 (-0.07;-0.01)†	-	-	0.84
MMP-9	1	-0.04 (-0.06;-0.02)†	-	-	0.46
(SD)	2	-0.04 (-0.06;-0.02)†	-	-	0.43
MMP-10	1	0.01 (-0.01;0.04)	-0.01 (-0.04;0.03)	0.04 (0.01;0.07)*	0.05
(SD)	2	0.01 (-0.01;0.04)	-0.01 (-0.04;0.02)	0.04 (0.01;0.07)*	0.03
TIMP-1	1	-0.02 (-0.04;0.01)	0.00 (-0.03;0.03)	-0.05 (-0.09;-0.01)†	0.03
(SD)	2	-0.02 (-0.04;0.01)	0.00 (-0.03;0.03)	-0.05 (-0.08;-0.01)†	0.04

Model 1: adjusted for age, sex (as appropriate), glucose metabolism status, systolic blood pressure, use of antihypertensive medication, prior cardiovascular disease, current smoking, waist, use of glucose- lowering medication (including insulin), estimated glomerular filtration rate, presence of albuminuria, total cholesterol, high density lipoprotein cholesterol, Ln triglycerides, use of lipid- modifying medication
Model 2: model 1 + use of renin-angiotensin-system inhibitors
*P value *< 0.05, †<0.01*

Supplemental table S7: Associations of serum MMP's and TIMP-1 levels with Ln LA volume index, in the population without outliers (linear regression, n=655)

	Model	Overall β (95% CI)	Men β (95% CI)	Women β (95% CI)	$P_{interaction}$ sex and MMP/TIMP
MMP-1	1	0.01 (-0.02;0.03)	0.03 (-0.01;0.06)	-0.01 (-0.05;0.02)	0.07
(SD)	2	0.00 (-0.02;0.02)	0.02 (-0.01;0.05)	-0.02 (-0.05;0.01)	0.06
	3	0.01 (-0.02;0.03)	0.03 (0.00;0.07)	-0.01 (-0.05;0.02)	0.05
MMP-2	1	0.04 (0.01;0.06)†	-	-	0.30
(SD)	2	0.03 (0.01;0.06)*	-	-	0.23
	3	0.03 (0.01;0.05)*	-	-	0.23
MMP-3	1	-0.03 (-0.06;0.00)*	-	-	0.94
(SD)	2	-0.04 (-0.07;-0.01)*	-	-	0.86
	3	-0.04 (-0.07;-0.01)*	-	-	0.91
MMP-9	1	-0.04 (-0.06;-0.01)†	-	-	0.50
(SD)	2	-0.04 (-0.06;-0.02)†	-	-	0.53
	3	-0.03 (-0.06;-0.01)†	-	-	0.53
MMP-10	1	0.01 (-0.02;0.03)	-0.01 (-0.05;0.02)	0.03 (0.00;0.07)	0.05
(SD)	2	0.01 (-0.02;0.03)	-0.01 (-0.05;0.02)	0.04 (0.00;0.07)*	0.03
	3	0.01 (-0.01;0.04)	-0.01 (-0.04;0.02)	0.04 (0.01;0.08)*	0.03
TIMP-1 (SD)	1	-0.02 (-0.05;0.01)	-0.01 (-0.05;0.03)	-0.03 (-0.07;0.01)	0.44
	2	0.03 (-0.06;0.00)	-0.01 (-0.05;0.03)	-0.04 (-0.08;-0.01)*	0.23

Model 1: adjusted for age, sex (as appropriate), glucose metabolism status
Model 2: model 1 + systolic blood pressure, use of antihypertensive medication, prior cardiovascular disease, current smoking, waist, use of glucose- lowering medication (including insulin), estimated glomerular filtration rate, presence of albuminuria, total cholesterol, high density lipoprotein cholesterol, Ln triglycerides, use of lipid- modifying medication
Model 3: model 2 + TIMP-1
*P value *< 0.05, †<0.01*
